# Combining urine lipoarabinomannan with antibody detection as a simple non-sputum-based screening method for HIV-associated tuberculosis

**DOI:** 10.1371/journal.pone.0218606

**Published:** 2019-06-25

**Authors:** Hiba Younis, Isabell Kerschbaumer, Jee-Young Moon, Ryung S. Kim, Caroline J. Blanc, Tingting Chen, Robin Wood, Steven Lawn, Jacqueline M. Achkar

**Affiliations:** 1 Department of Medicine, Albert Einstein College of Medicine, Bronx, New York, United States of America; 2 Department of Epidemiology and Population Health, Albert Einstein College of Medicine, Bronx, New York, United States of America; 3 Desmond Tutu HIV Centre, Institute of Infectious Diseases and Molecular Medicine, University of Cape Town, Cape Town, South Africa; 4 Department of Clinical Research, Faculty of Infectious and Tropical Diseases, London School of Hygiene and Tropical Medicine, London, United Kingdom; 5 Department of Microbiology and Immunology, Albert Einstein College of Medicine, Bronx, New York, United States of America; Rutgers Biomedical and Health Sciences, UNITED STATES

## Abstract

**Background:**

Simple methods for the accurate triaging and screening of HIV-associated tuberculosis (TB) are urgently needed. We hypothesized that combining serum antibody with urine lipoarabinomannan (U-LAM) detection can improve the detection of HIV-associated TB.

**Methods:**

We performed a case-control study with sampling from a prospective study of South African HIV-infected subjects who were screened for TB prior to initiating antiretroviral therapy. Sera from all available TB cases (n = 74) and randomly selected non-TB controls (n = 30), all tested for U-LAM, sputum microscopy, GeneXpert, and cultures, were evaluated for antibodies to LAM and arabinomannan (AM). Diagnostic logistic regression models for TB were developed based on the primary test results and the additive effect of antibodies with leave-one-out cross-validation.

**Results:**

Antibody responses to LAM and AM correlated strongly (p<0.0001), and IgG and IgM reactivities were significantly higher in TB than non-TB patients (p<0.0001). At 80% specificity, the target specificity for a non-sputum-based simple triage/screening test determined by major TB stakeholders, combining U-LAM with IgG detection significantly increased the sensitivity for HIV-associated TB to 92% compared to 30% for U-LAM alone (p<0.001). Sputum microscopy combined with IgG detection increased sensitivity to 88% compared to 31% for microscopy alone, and Xpert with IgG increased sensitivity to 96% and 99% compared to 57% for testing one, and 70% for testing two sputa with Xpert alone, respectively.

**Conclusion:**

Combining U-LAM with serum antibody detection could provide a simple low-cost method that meets the requirements for a non-sputum-based test for the screening of HIV-associated TB.

## Introduction

Active tuberculosis (TB) is the leading cause of both death from a single pathogen and death in HIV co-infected individuals [1]. Of the estimated 10.4 million people who developed TB in 2016, 10% were HIV co-infected; and of the close to 1.7 million who died of the disease that year, nearly 0.4 million had HIV-associated TB [1]. Difficulty in the rapid identification of HIV-associated TB often delays treatment and negatively affects patient outcomes, particularly in resource poor settings [2]. Evidence for this is supported by post-mortem data showing that 40% of HIV-related deaths in resource-limited settings are due to TB—almost half of which were undiagnosed at the time of death [3]. The current gold standards for TB diagnosis are mycobacterial sputum cultures and nucleic acid amplification tests (NAATs) such as the GeneXpert^®^ (commonly referred to as "Xpert"; Cepheid Inc., Sunnyvale, California) [4]. However, cultures take weeks to become positive, and both cultures and NAATs can have limited sensitivity in HIV-infected individuals who often have disseminated or extrapulmonary manifestation [2, 5–7]. Although the ability to obtain rapid results with NAATs is a major benefit, these tests require technology not available in many resource poor settings or at the community healthcare level [8]. Other methods currently used include sputum microscopy, which, although rapid and low-cost, has a limited sensitivity of typically below 50% in HIV-infected individuals [2, 5, 8, 9]. Thus, establishing a diagnosis of HIV-associated TB can be challenging and is further complicated by the myriad of other HIV-associated opportunistic diseases that can present with similar signs and symptoms. Furthermore, up to over 30% of HIV-infected individuals living in high TB incidence settings have undiagnosed TB when screened prior to initiation of antiretroviral therapy (ART) [9]. Therefore, as recently emphasized at a major TB stakeholder meeting, both rapid, low-cost screening as well as triage tests for HIV-associated TB are urgently needed [8]. Ideally, such tests should be non-sputum-based, device-free, and usable at the community health care level.

Additional diagnostic tests for HIV-associated TB are based on the detection of the mycobacterial cell wall glycolipid lipoarabinomannan (LAM) in the patients’ urine, as reviewed by Shah et al. [10]. These urinary LAM (U-LAM) tests are available in form of an enzyme-linked immunosorbent assay (ELISA; Clearview TB-ELISA, Alere, Waltham, MA, USA) or a simple lateral flow (dipstick) format (Determine TB-LAM, Alere). It has long been known that LAM can be isolated from *M*. *tuberculosis* cultures [11, 12]. Therefore, its detection in the urine could be either due to the presence of mycobacteria in the urinary tract, or, because of its small size of approximately 19 kDa, due to glomerular filtration of non-antibody bound LAM from the blood into the urine of TB patients with high mycobacterial burden [13]. It is thus not surprising that U-LAM detection has been predominantly associated with both renal as well as disseminated TB, but less with locally confined pulmonary disease [13–15]. These test characteristics are clinically relevant because extrapulmonary/disseminated TB occurs more frequently in late stage HIV patients with low CD4 counts and is particularly challenging to diagnose [8]. Pooled data from several studies show an overall test sensitivity and specificity of 45–47% and 92–96%, respectively, which change to 56% and 90%, respectively, in patients with CD4 counts <100 cells/ul [10]. Thus, the use of the lateral flow format has been endorsed by the World Health Organization (WHO) for HIV-infected patients with suspected TB and CD4 counts under 100 cells/μl [16]. Given that HIV-infected individuals frequently develop TB at CD4 counts above 100 cells/ul, the clinical value of the U-LAM test is limited if used alone [10]. The combination of the U-LAM test with other diagnostics, such as sputum smear microscopy and Xpert, can significantly increase the detection of HIV-associated TB and reduce mortality in HIV-infected TB suspects with low CD4 counts [15, 17]. However, due to the required equipment, these combinations are of limited value in many remote and low resource settings and at the level of community healthcare workers, leaving a need for simple, rapid, and non-sputum-based triage as well as screening tests for HIV-associated TB [2, 8].

Immunological methods such as TB serodiagnostic assays are based on detecting antibodies (Abs) to mycobacterial antigens, as reviewed by Steingart et al. [18]. Similar to U-LAM-based tests, the benefits of serologic assays are their independence of sputum analysis, potential for detection of all forms of TB, and suitability for rapid, simple, low-cost format development (e.g. dipstick test). Thus far, commercially available TB serodiagnostic assays have shown limited and variable sensitivity and specificity (0 to 100% and 31 to 100%, respectively), largely dependent on study population, settings, antigens, and diagnostic cut-off values used [18]. We and others have previously shown that TB patients have significantly higher Abs to LAM and/or its polysaccharide component arabinomannan (AM) than controls [19–24]. While most groups focused on HIV uninfected patients, we found that IgG responses to AM and LAM were significantly higher in both HIV uninfected and HIV-infected patients with TB compared to respective controls [19]. Although HIV co-infected patients had lower Ab titers than HIV uninfected TB patients, the detection of such Abs could have adjunctive value in identifying HIV-associated TB. We hypothesized that combining U-LAM detection with that of serum Abs to LAM or its non-lipidated counterpart AM can significantly increase the sensitivity for HIV-associated TB compared to U-LAM detection alone. Our primary objective was the assessment of serum Abs to LAM/AM and its adjunctive value to U-LAM detection in HIV-infected subjects who were screened for TB [25]. As a secondary objective, we evaluated whether Ab detection has adjunctive value to other available point-of-care (POC) tests such as sputum smear microscopy and Xpert.

## Materials and methods

### Study design and subjects

**Fig 1:**
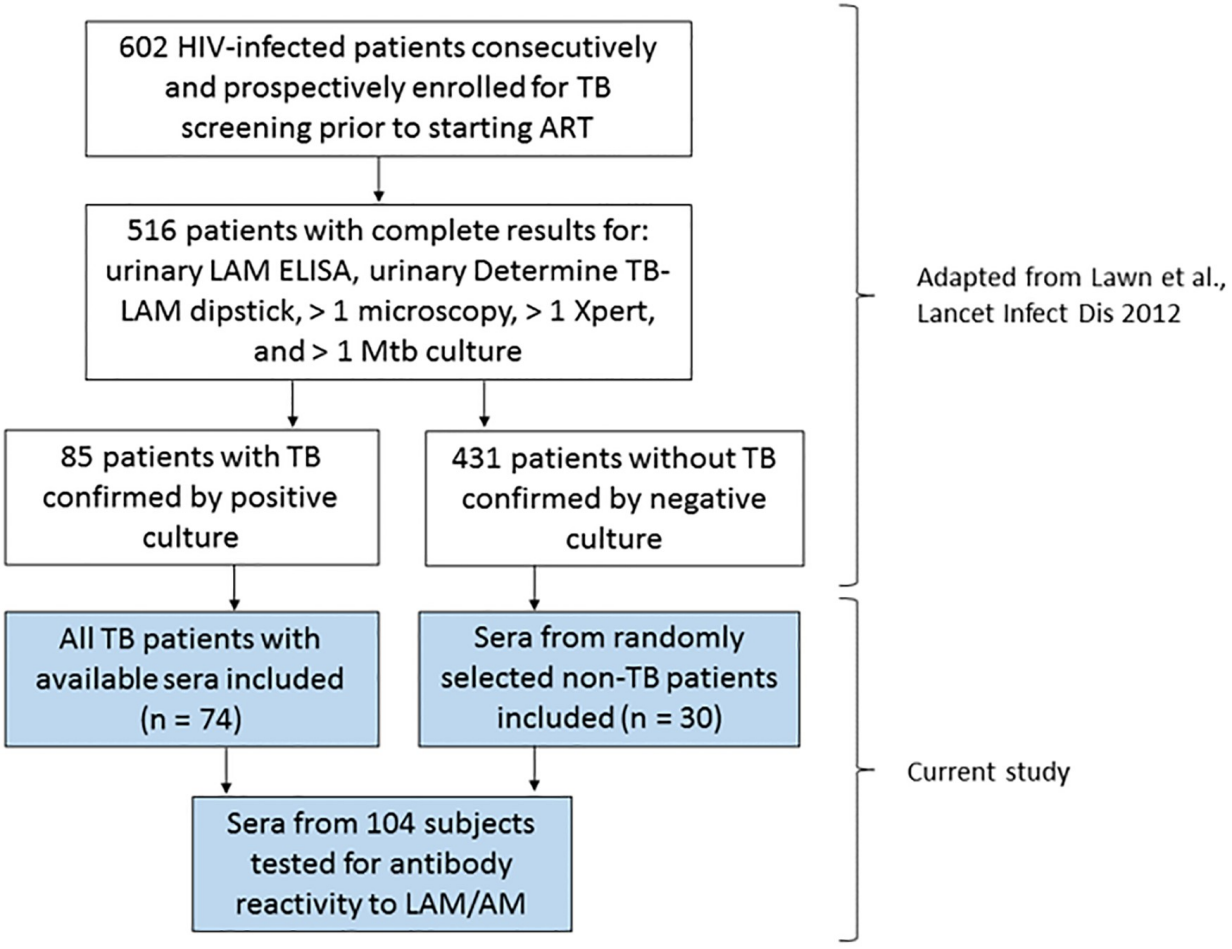
Study flow diagram for parent [25] and current study. ART: antiretroviral therapy.

This was a case-control study in which sera were obtained from a study of HIV-infected patients who were previously prospectively and consecutively enrolled to assess the clinical utility of U-LAM detection for the detection of TB prior to initiation of antiretroviral treatment (ART; [25]). The parent study was in compliance with the standards for reporting of diagnostic accuracy (STARD; [26]) and enrolled 602 ART naive adult subjects, at least 18 years old and without a concurrent TB diagnosis, from March 2010 to April 2011 from the ART service in Gugulethu township, Cape Town, South Africa, a setting with a high TB incidence. Recruitment criteria, collection of urine and serum samples, and diagnostic tests were performed as described, and complete test results were available for 516 subjects [25]. In the parent study, 85 sputum culture-positive TB patients had results available for all diagnostic tests. Of these, sera were available for 74 subjects and all were included in our study (Fig 1). In the parent study, based on negative work-up, TB was ruled out in over 431 subjects. Because including of all of these non-TB subjects was not feasible for our study, 30 subjects without a history of TB were randomly chosen to be included as controls. This number was determined to be sufficient because in our prior US-based pilot study with biologically independent samples Ab response to AM were significantly higher in 14 HIV-infected TB patients compared to 38 HIV-infected controls (p < 0.01) [19]. Based on these results our power to detect differences in Ab reactivity with 74 TB+ and 30 non-TB subjects at a significance level < 0.05 exceeded 0.8. Sera were evaluated for Ab responses to LAM and AM to assess the additive diagnostic value of Ab to U-LAM detection and other POC tests as performed in the parent study. Approval for research on human subjects was obtained from the research ethics committees of the University of Cape Town, South Africa, the London School of Hygiene & Tropical Medicine, UK, and the institutional review board of the Albert Einstein College of Medicine, NY, USA. Written informed consent was obtained from all subjects prior to enrollment.

### Mycobacterial antigen preparations

We used LAM and AM, components of the mycobacterial cell wall and capsule, respectively [27]. LAM isolated from the *M*. *tuberculosis* strain H37Rv was obtained from the Biodefense and Emerging Infections Research Resources Repository (BEI; Manassas, VA). Capsular AM was isolated and purified from *M*. *tuberculosis* strain H37Rv as described [19, 27]. Although Ab responses to AM and LAM correlated strongly and significantly among TB cases with or without HIV in our prior studies [19, 27], IgG responses to AM, in contrast to LAM, were significantly different between smear-positive versus smear-negative TB patients, suggesting that AM could be a more sensitive marker for mycobacterial burden [19]. For this reason, and also because i) LAM from BEI was only available in small quantities, and ii) the isolation of AM in larger quantities is less time-consuming than LAM, we planned to use AM in lieu of LAM for the assays after confirming the strong and significant correlation with 20 randomly chosen samples from subjects of this study.

### U-LAM detection, microscopy and Xpert tests

The presence of LAM in the urine samples was determined in the parent study and quantified using the Determine TB-LAM dipstick test and the commercially available Clearview TB enzyme-linked immunosorbent assay (ELISA) as per manufacturer’s instructions [25]. Sputum samples were processed and evaluated for acid-fast bacilli by fluorescent microscopy and *M*. *tuberculosis* by Xpert according to standard procedures and protocols [25].

### Ab detection assays

ELISAs were performed as described [19, 27]. Briefly, 96-well plates (Maxisorp nunc, Thermo Fisher Scientific) were coated with AM and initially also LAM (for comparisons between AM and LAM) at 10 *μ*g/ml and blocked overnight with 3% BSA in 0.1% PBS-T. Serum samples, diluted 1:50, were added in duplicates to the antigen-coated wells, and the bound Abs detected with either alkaline phosphatase (AP)-conjugated protein A for the detection of IgG, goat anti-human IgM-AP or goat anti-human IgA-AP (1:1000; Sigma, St Louis, MO). Optical densities (OD) were measured at 405 nm. Negative and positive controls were processed in duplicates as described above, except that serum was replaced, respectively, with diluted blocking buffer for negative controls or the murine monoclonal Ab CS35 (1:200) known to recognize AM and LAM from various mycobacterial strains as a positive control, followed by detection with anti-mouse IgG3-AP (1:1000; Sigma). To assure reproducibility of data, each assay was repeated on two separate days. Although the person performing the assays was not blinded to the TB diagnosis of the subjects, she was at the time of performing the assays blinded to the diagnostic test results associated with each sample.

### Statistical analysis

Statistical analysis was performed using GraphPad Prism, version 7 (GraphPad Inc., San Diego, CA) and R, version 3.3.2 (R Foundation, Vienna, Austria). Demographics, clinical characteristics and diagnostic test results were compared between patient groups using Fisher’s exact test for categorical variables and the Mann-Whitney test for continuous variables. Ab responses were compared between groups by Mann-Whitney and correlated within groups by Spearman rank correlation tests. Data analysis focused on U-LAM detection results with the POC dipstick format. Diagnostic models for TB+ were developed and their accuracy measured with *M*. *tuberculosis* positive culture as the gold standard. We first diagnosed patients to be TB+ if the primary test (e.g. U-LAM) result was positive. Then, for patients whose primary test results were negative, we built logistic regression models to diagnose TB status using Abs (IgG, IgM and IgA to AM/LAM) as covariates. In the Ab only models, we built a logistic regression model to diagnose all patients regardless of other diagnostic test results. The target product profile for a non-sputum-based simple low-cost triage test for TB was set at a specificity between 70 to 80% at a recent major TB stakeholders meeting, including the World Health Organization (WHO) and the Centers for Disease Control (CDC) [8]. We therefore evaluated sensitivity at 80% specificity. For each diagnostic model we considered clinically relevant (sensitivity >85% at a specificity of 80%), and because we considered multiple diagnostic models, we performed a final two-loop leave-one-out cross-validation to avoid overfitting of estimated receiver operating characteristic (ROC) curves [28, 29]. On each *n*-1 subject, we performed inner-loop cross-validation (with *n*-2 subjects) to estimate sensitivity at 80% specificity, and repeated the selection process *n* times to calculate the average mean sensitivity. While this method does not replace the validation on an independent sample set, it can provide an unbiased estimate of accuracy measures under mild assumptions. To compare the sensitivity of the diagnostic model, e.g. combining U-LAM with IgG detection and that with U-LAM alone, we used generalized linear mixed effects model taking into account the paired diagnoses for each sample.

## Results

### Demographics and clinical variables

No significant differences between TB patients and controls were observed for demographics as well as CD4 counts. Although many non-TB controls were symptomatic and the groups were not significantly different in the occurrence of fever, TB patients had a significantly more other TB-associated symptoms and other clinical characteristics such as cough, shortness of breath, sweats, weight loss and chest X-ray abnormalities (Table 1). Of the TB+ subjects, 22 (30%) were U-LAM+ by dipstick and 21 by ELISA as determined in the parent study [25]. U-LAM+ compared to U-LAM- TB+ subjects (as per U-LAM dipstick) were slightly but significantly younger and had significantly lower CD4 counts; otherwise the two groups were similar in demographics and clinical presentation (Table 2).

**Table 1:** Demographics and clinical characteristics of HIV+ subjects stratified by TB status.

Characteristics	TB Patients (*n* = 74)	Non-TB Patients (*n* = 30)	P*
Male sex (%)	27 (36)	11 (37)	1
Age, median years [25%ile, 75%ile]	33 [28, 38]	31 [27, 36]	0.19
Current smoker (%)	15 (20)	6 (20)	1
Previous TB (%)	14 (19)	0	0.009
CD4, median cells/mm^3^ [25%ile, 75%ile]	138 [60, 204]	135 [80, 201]	0.94
Abnormal CXR^a^ (%)	53 (75)	9 (32)	0.0002
Initial sputum AFB smear positive (%)	19 (26)	0	0.001
Two sputa combined AFB^b^ smear positive (%)	23 (31)	0	0.0002
Initial sputum GeneXpert positive (%)	42 (57)	0	<0.0001
Two sputa combined GeneXpert positive (%)	52 (70)	0	<0.0001
U-LAM dipstick+ (%)	22 (30)	0	0.0003
U-LAM ELISA+ (%)	21 (28)	0	0.0004
TB home contact	1 (1)	3 (10)	0.07
Symptoms present at evaluation:			
Cough	48 (65)	10 (33)	0.005
Hemoptysis	2 (3)	1 (3)	1
Shortness of breath	32 (43)	3 (10)	0.001
Night sweats	40 (54)	9 (30)	0.03
Fever	22 (30)	7 (23)	0.63
Weight loss	62 (84)	18 (60)	0.019

*P-values for categorical variables are given using the Fisher’s exact test and for continuous variables using the Mann-Whitney *U* test;

^a^: CXR: chest X-ray;

^b^: AFB: acid fast bacilli

**Table 2:** Demographics and clinical characteristics of HIV+ TB patients stratified by U-LAM dipstick result.

Characteristics	U-LAM+ TB Patients (*n* = 22)	U-LAM- TB Patients (*n* = 52)	P*
Male sex (%)	5 (23)	22 (42)	0.124
Age, median years [25%ile, 75%ile]	30 [26, 34]	34 [29, 41]	0.016
Current smoker (%)	2 (9)	13 (25)	0.205
Previous TB (%)	4 (18)	10 (19)	1
CD4, median cells/mm^3^ [25%ile, 75%ile]	36 [22, 131]	176 [105, 216]	0.0001
Abnormal CXR^a^ (%)	15 (71)	38 (76)	0.768
Initial sputum AFB^b^ smear positive (%)	11 (50)	8 (15)	0.003
Two sputa combined AFB smear positive (%)	11 (50)	12 (23)	0.030
Initial sputum GeneXpert positive (%)	19 (86)	23 (44)	0.0008
Two sputa combined GeneXpert positive (%)	20 (91)	32 (62)	0.013

*P-values for categorical variables are given using the Fisher’s exact test and for continuous variables using the Mann-Whitney *U* test;

^a^: CXR: chest X-ray;

^b^: AFB: acid fast bacilli

### Correlation between Ab responses to LAM and AM

**Fig 2:**
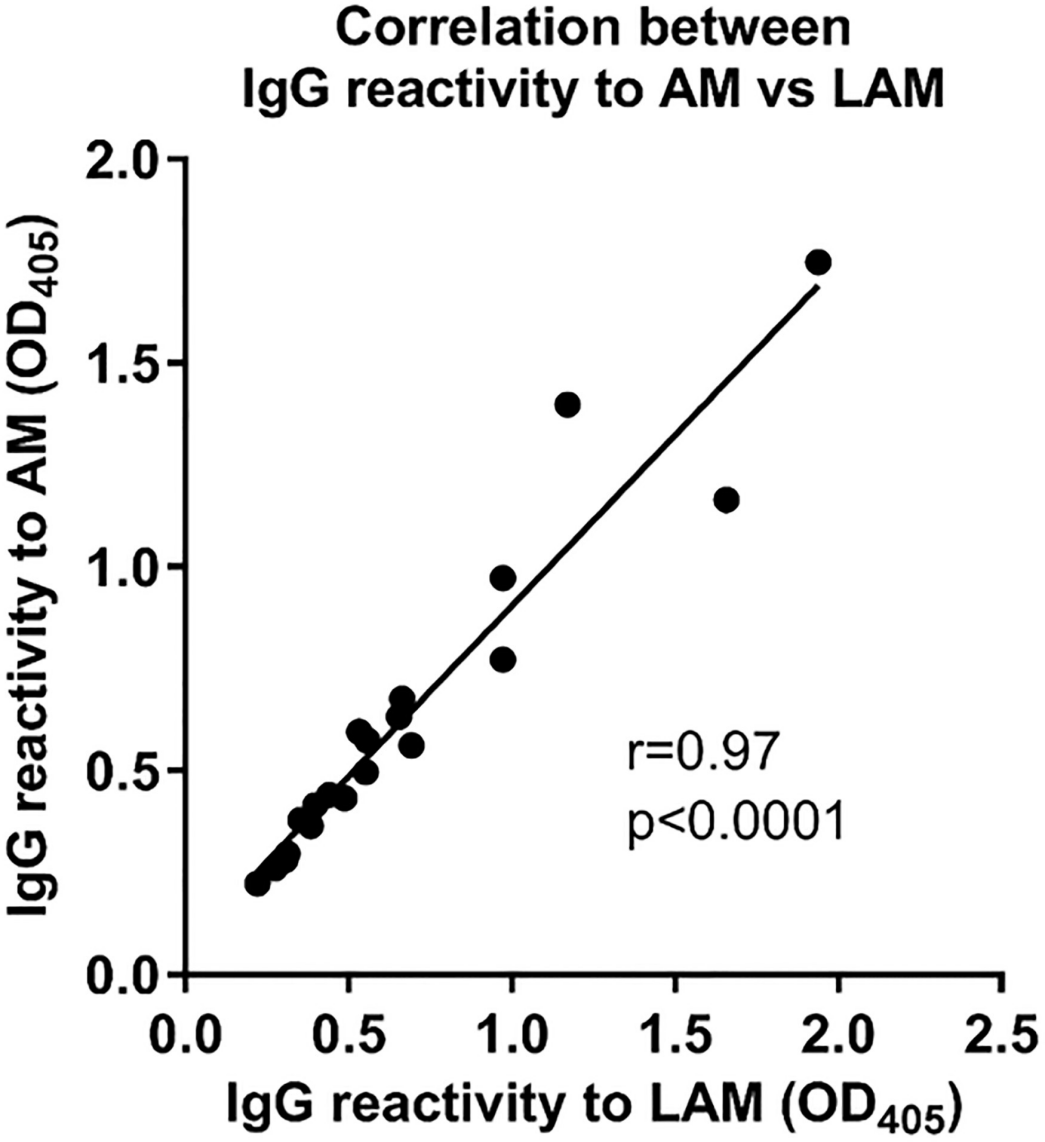
Correlation between IgG response to *M*. *tuberculosis* capsular arabinomannan (AM) and cell wall lipoarabinomannan (LAM) in HIV-infected TB patients. Spearman rank correlation.

Consistent with our prior data [19, 27], IgG responses to LAM correlated strongly and significantly with those to AM (r = 0.97, p<0.0001; Fig 2). Thus, for practical reasons, we evaluated Ab responses using AM.

### Ab responses to AM by TB and U-LAM status

**Fig 3:**
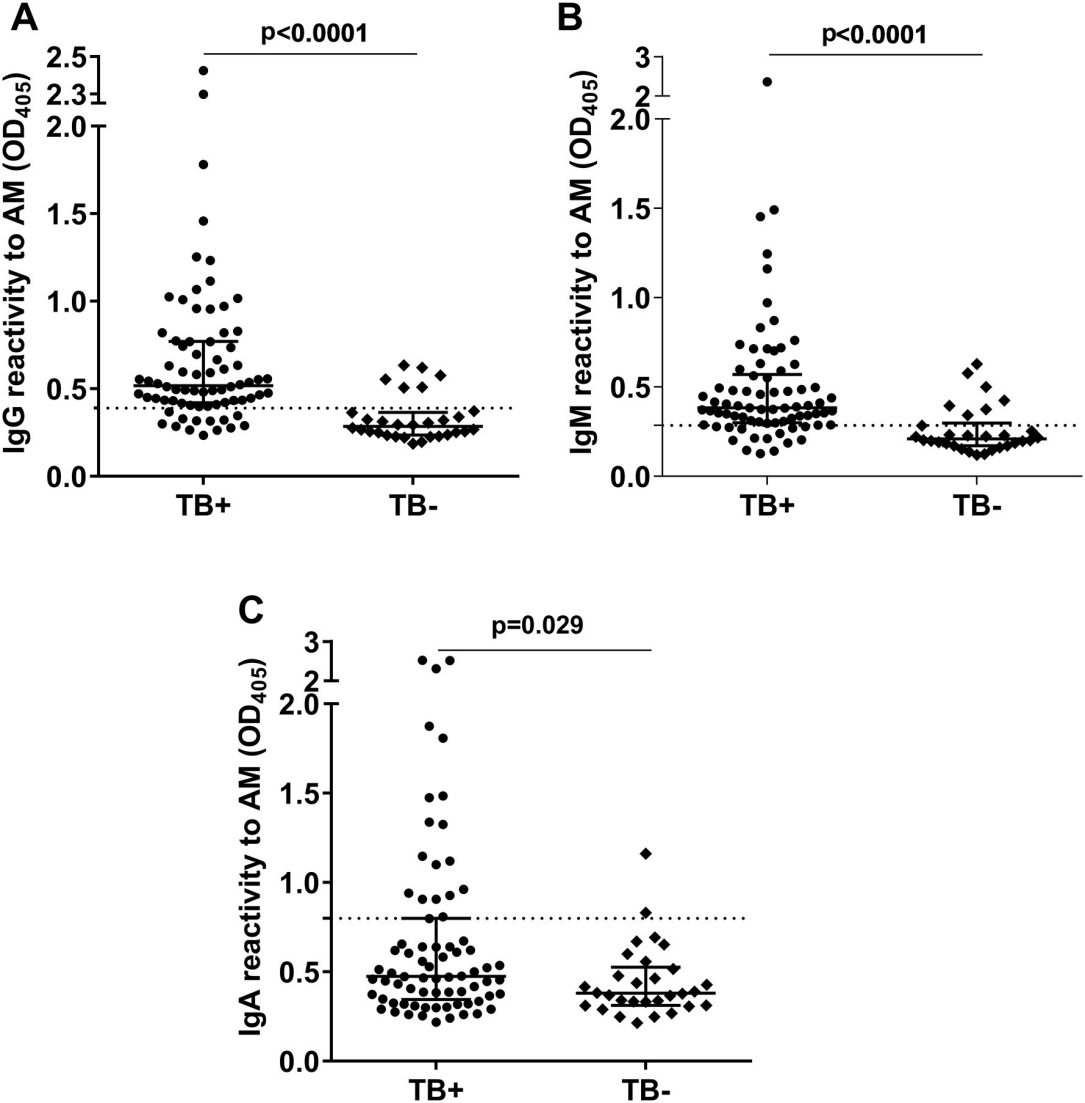
Comparison of antibody responses to AM between TB+ and TB- subjects. **(A)** IgG to AM; **(B)** IgM to AM; **(C)** IgA to AM. Lines and error bars represent medians with interquartile ranges. Mann-Whitney *U* test was used for statistical comparisons. Dotted lines represent Ab level cut-off determined by receiver operating curve (ROC) at specificity of 80% showing a sensitivity for TB of 82%, 77%, and 46% for IgG, IgM, and IgA, respectively.

**Fig 4:**
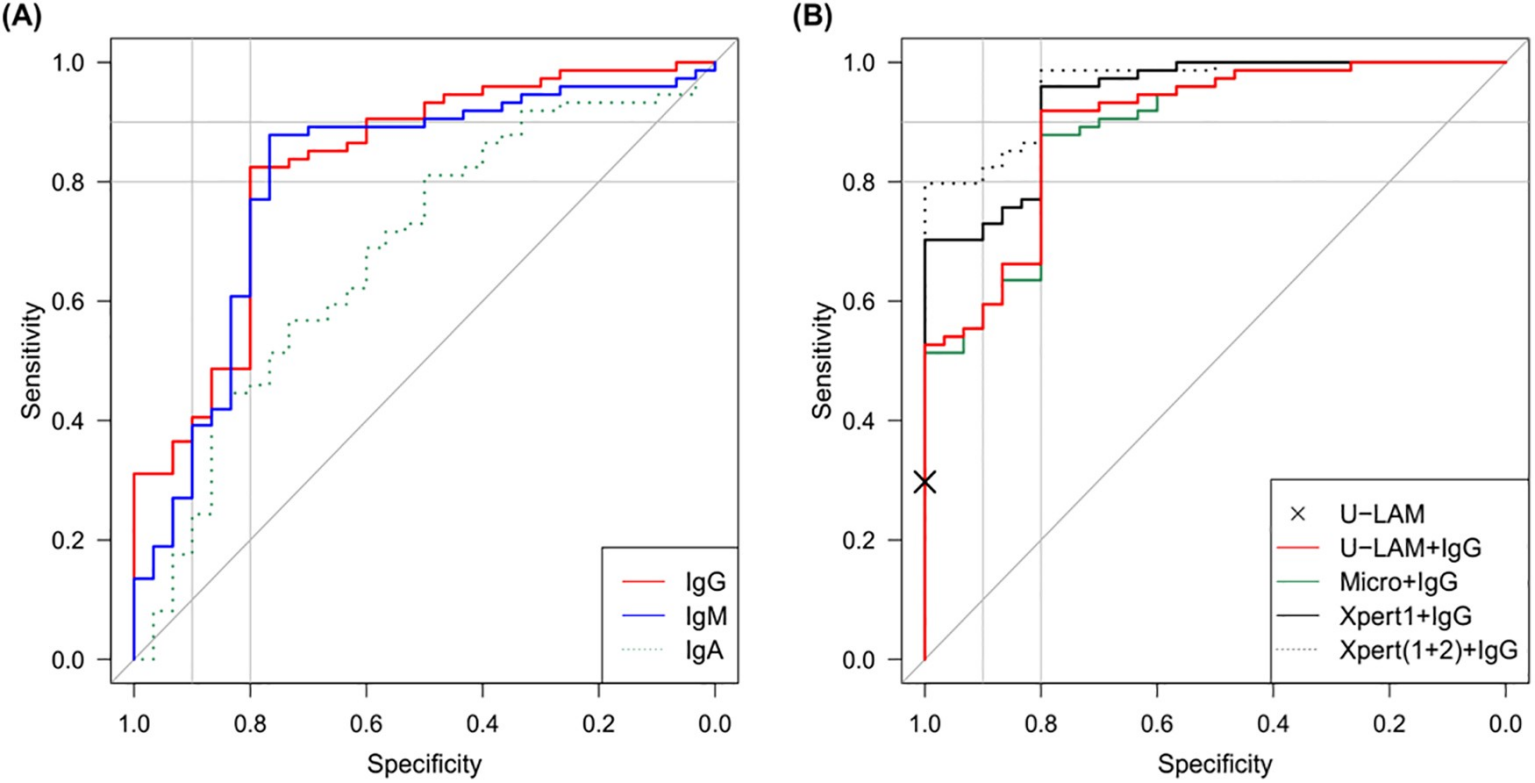
Receiver operation characteristic (ROC) curves for detection of TB with M. tuberculosis culture as reference gold standard. **(A)** IgG, IgM, and IgA to AM; **(B)** IgG to AM combined with other rapid diagnostic tests. ROC curves include cross-validation.

**Fig 5:**
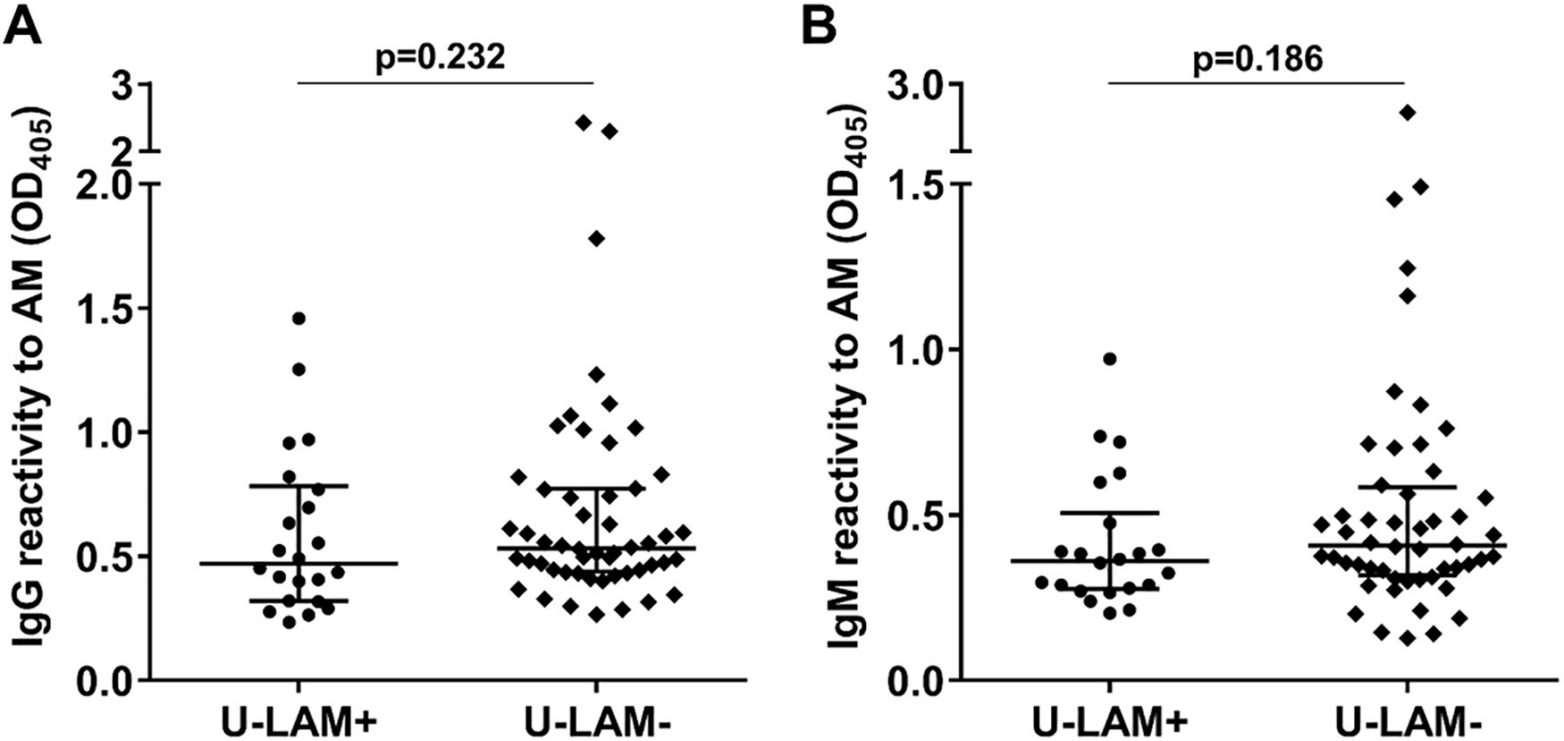
Comparison of IgG response to AM between U-LAM+ and U-LAM- TB+ subjects. **(A)** IgG to AM; **(B)** IgM to AM. Lines and error bars represent medians with interquartile ranges. Mann-Whitney *U* test was used for statistical comparisons.

IgG, IgM, and IgA responses to AM were significantly higher in TB+ than non-TB subjects, with IgA discriminating the groups less (p = 0.029) than IgG or IgM (p<0.0001; Fig 3). Because our primary objective was the assessment of non-sputum-based methods for triage testing for HIV-associated TB, we set our target specificity at 80%, a number identified to be optimal for such a test [8]. At this specificity, the sensitivity for serum IgG to AM was 82%, for IgM 77%, and for IgA to AM 46% (Figs 3 and 4A). Combining the detection of Ab isotypes to AM did not increase sensitivity beyond that of IgG alone when adjusting cut-off values to maintain a specificity of 80%. Importantly, there was no significant correlation between CD4 counts and IgG (r = 0.06, p = 0.59), IgM (r = 0.06, p = 0.62), or IgA titers to AM (r = -0.05, p = 0.68), demonstrating that Ab detection was not influenced by the level of immune suppression. Similar to proportions reported for the larger parent study [25], the sensitivities of the U-LAM dipstick and ELISA for TB were 30% and 28%, respectively, with a specificity of 100% for both (Table 1). A non-significant trend towards higher Ab responses to AM was observed for IgG and IgM but not IgA in the U-LAM- compared to the U-LAM+ TB+ subjects (Fig 5), supporting the adjunctive value of IgG when combined with U-LAM detection.

### Diagnosis of TB by combining Ab with U-LAM detection and other POC tests

**Fig 6:**
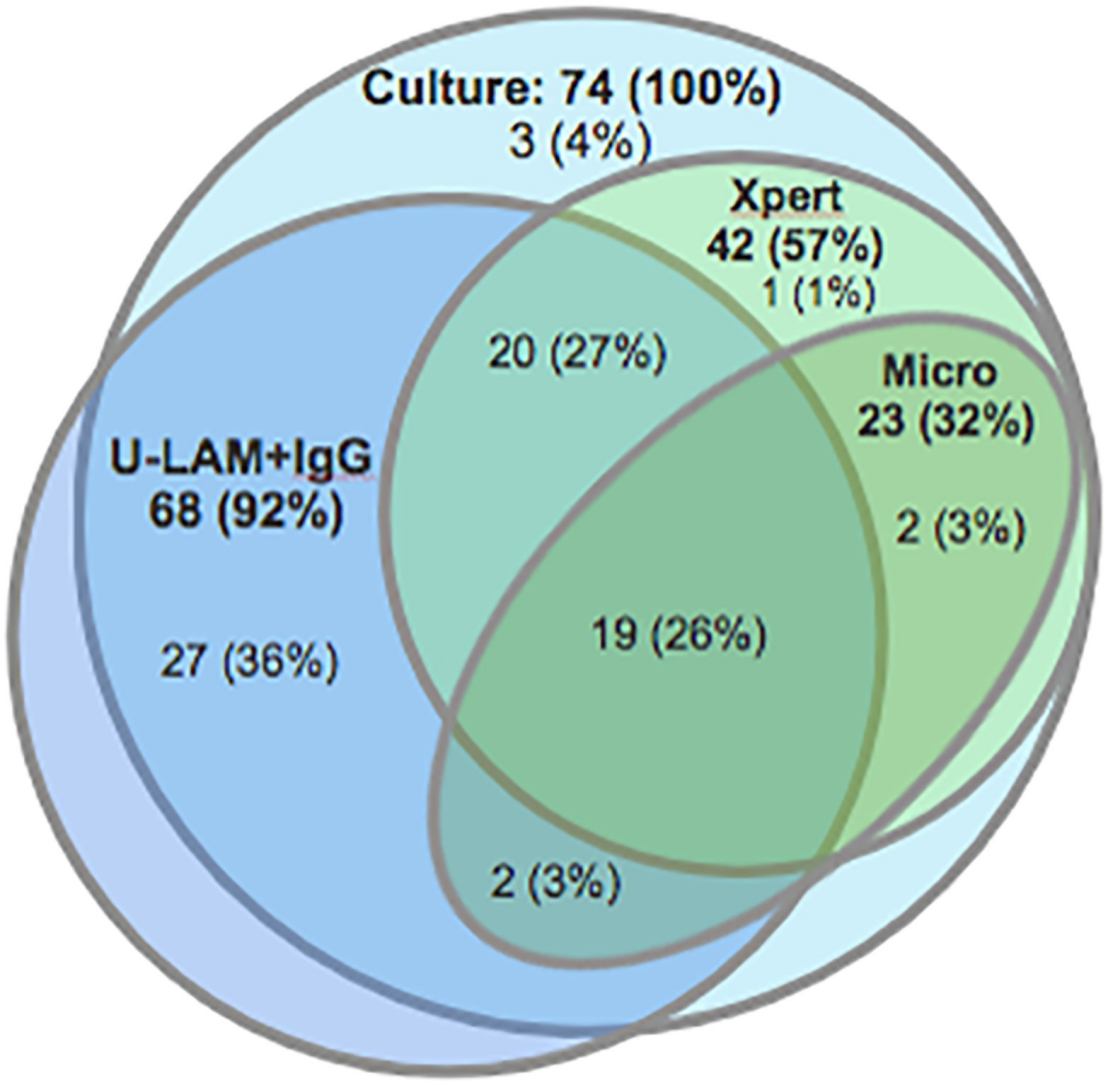
Venn diagram showing proportions of TB patients diagnosed by different test modalities relative to those diagnosed by mycobacterial culture (n = 74 (100%)). Numbers in bold fonts represent patients testing positive by the respective test modalities while those in regular fonts show numbers and proportions of patients exclusively diagnosed by this/these compartment(s). Culture = *M*. *tuberculosis* culture positive; Xpert = initial sputum GeneXpert positive; Micro = two sputum smears for acid fast bacilli positive; and U-LAM+IgG = urinary LAM detection by urine dipstick test and/or IgG to AM positive. Of note, the dark blue fraction of the U-LAM+IgG circle extending outside the *M*. *tuberculosis* culture positive circle represents anti-AM IgG positive, culture negative subjects.

Next, we investigated the adjunctive value of Abs in detecting HIV-associated TB at a specificity of 80%. For patients whose primary test results (e.g. U-LAM dipstick) were negative, we built a logistic regression model using up to all three Ab isotypes to AM (always including IgG) as covariates. In this model, which included cross-validation, combining U-LAM with IgG detection significantly increased the sensitivity for HIV-associated TB to 92% compared to 30% for U-LAM alone (p<0.001; Table 3 & Figs 4 and 6). While sputum microscopy (two sputa) combined with IgG detection increased sensitivity to 88% compared to 31% for microscopy alone, combining Xpert with IgG increased sensitivity to 96% and 99% compared to 57% for testing one, or 70% for testing two sputum samples with Xpert alone, respectively (Table 3 & Figs 4 and 6). Combining two or more diagnostic methods with IgG detection did not lead to further meaningful increases in sensitivity.

**Table 3:** Performances of diagnostic models for HIV-associated TB.

Diagnostic model	SS at SP = .8
**U-LAM**^a^**+ or IgG**^b^**+**	**0.92**
**Micro(1+2)+ or IgG**^b^**+**	**0.88**
**Xpert(1)+ or IgG**^b^**+**	**0.96**
**Xpert(1+2)+ or IgG**^b^**+**	**0.99**
U-LAM^a^+ or Micro(1+2)+ or IgG^b^+	0.95
U-LAM^a^+ or Xpert(1)+ or IgG^b^+	0.96
U-LAM^a^+ or Xpert(1+2)+ or IgG^b^+	0.99

SS: Sensitivity; SP: Specificity;

^a^: urinary lipoarabinomannan dipstick test (Determine TB-LAM, Alere);

^b^: IgG to AM at cut-off OD > 0.39

## Discussion

A simple non-sputum-based screening POC test for HIV-associated TB, suitable for use in resource-limited settings, is a high priority diagnostic need [8]. Ideally such a test would be usable for both screening for TB in HIV clinics and triaging of symptomatic HIV-infected patients in community healthcare centers to identify those individuals that should undergo further confirmatory testing for HIV-associated TB. We here demonstrate that combining the detection of U-LAM with that of serum Abs to LAM or AM could provide such a method. With 92% sensitivity at a specificity of 80% for HIV-associated TB this combination, if validated in other studies, holds promise that it could meet the desired target product profile for a simple triage/screening test determined by major TB stakeholders including the WHO and CDC [8]. Since Abs can be detected with simple lateral flow (dipstick) formats, similar to the already available U-LAM POC test, a combination of these tests would be device-free and suitable for use of healthcare workers in settings without laboratory infrastructure. It could thus fill a gap in the urgent needs for additional TB diagnostics.

Improving the detection of TB is important in both symptomatic and asymptomatic HIV-infected individuals. TB preventative therapy is recommended by the WHO for all HIV-infected individuals living in TB endemic regions [30]. Ruling out TB prior to initiation of preventative therapy is critical because single or dual drug therapy could lead to drug-resistance when given to patients with disease. Furthermore, subclinical TB, an asymptomatic form of disease in HIV co-infected patients, occurs in up to over 10% of TB cases detected during screening prior to ART and can be associated with high mortality rates if left untreated for even a few months [31–36]. Because it is typically sputum microscopy and Xpert negative, its detection would require non-sputum-based tests. Given that our study population consisted of subjects being screened for TB prior to initiation of ART, our data hold promise that over 90% of HIV-associated TB cases could be identified by combining U-LAM with anti-AM/LAM IgG detection.

A simple triage or screening test with high sensitivity and moderate specificity would allow to focus on only the test positive subjects for confirmatory testing. In that respect, C-reactive protein (CRP), a non-specific inflammatory protein with POC potential for TB, has shown a high sensitivity of up to 98% albeit at a lower specificity of below 60% in the triaging for TB in symptomatic [37, 38], and a sensitivity of 89% with a specificity of 72%, respectively, in the screening of asymptomatic HIV-infected individuals [39]. Although detection of Abs to *M*. *tuberculosis* bear the risk of reduced specificity due to cross-reactivity with antigens of non-tuberculous mycobacteria, they have the potential to be more specific than inflammatory biomarkers. Consistent with this notion, sensitivities assessed at the 80% specificity level in our study demonstrate a more favorable profile than that of CRP. Due to limited available serum volumes, we focused on investigating Ab responses to LAM/AM in combination with U-LAM detection. It is conceivable that by either adding other easily and rapidly detectable and accurate serum biomarkers, some of which have been recently reported by us and others [40–46], and/or by enhancing the sensitivity of the current U-LAM detection via approaches recently reported [47–49], the sensitivity of combined methods for HIV-associated TB could be enhanced to over 95%.

The significant difference between anti-AM IgG reactivity in HIV-infected TB versus non-TB patients is in accordance with results from our previous studies with TB and non-TB patients living in the US [19]. This is an important observation because the US represents a region of low TB incidence while South Africa has one of the highest TB incidence rates globally [1]. The validation of our US with the South African data shown here supports the notion that despite the known limitations of Ab detection for TB diagnosis [18], it could, when used in combination with other simple tests, be a useful adjunctive biomarker for the screening or triaging of HIV-associated TB. Although less observed in this study, IgM is known for lower accuracy than other isotypes in TB serology studies [18, 19]. The high titers of IgM to AM in many of the South African HIV-infected TB patients in our current study could indicate high rates of new *M*. *tuberculosis* infection rather than reactivation of remote infection. Such hypothesis warrants further exploration but is supported by both significantly increased IgM responses to AM in Ugandan TB household contacts who convert their tuberculin skin-test versus those who do not (unpublished data by our group) and the known high rates of TB due to new *M*. *tuberculosis* infection in HIV-infected adults living in TB endemic regions [50–52]. The strong correlation between Ab responses to LAM and its non-lipidated counterpart AM in this study is also consistent with our prior human data [19, 27]. It confirms that LAM and AM can be used interchangeably and irrespective of HIV infection status in serology studies. It further provides the option to test for reactivity to synthetically generated AM oligosaccharide fragments in future studies. These are now available and, compared to native polysaccharide fragments, would have the advantage of greater product consistency [27].

Another important result was the lack of correlation between IgG responses and CD4 counts, assuring that the adjunctive diagnostic value of Ab detection was not limited to a subset of HIV-infected individuals. Although we believe that combining U-LAM with Ab detection would be the most suitable method for triaging or screening in resource-limited settings, a combination of other diagnostic tests with Ab detection showed also enhanced sensitivities for TB. Sputum microscopy combined with IgG detection increased sensitivity to 88% compared to 31% for microscopy alone, and Xpert with IgG increased sensitivity to 96% and 99% compared to 57% for testing one, and 70% for testing two sputum samples with Xpert alone, respectively. Depending on the setting, these results, if validated in larger studies, could have important clinical implications.

Limitations of our study include a moderate sample size and the lack of validation in an independent sample set. We thus applied leave-one-out cross-validation to avoid overfitting of our diagnostic models [28, 29]. Furthermore, our study limited selection bias by using sera from prospectively and consecutively enrolled HIV-infected subjects of a study that followed the STARD guidelines [25]. Comparison against the gold standard for TB, automated liquid culture of sputum done in an accredited laboratory [25], further support the accuracy of our results. Nevertheless, larger studies, using the by us determined cut-off values from positive Ab reactivity, are needed to validate our results. Because our subjects were HIV-infected outpatients being screened for TB prior to ART initiation regardless of symptoms, assessing the triage value of our method would also require the inclusion of symptomatic HIV-infected individuals with other respiratory diseases.

In conclusion, the combination of U-LAM and serum Ab detection could provide a simple non-sputum-based screening, and possibly also triage method, to identify those HIV-infected individuals who should undergo further confirmatory testing for HIV-associated TB. Larger studies are needed to validate our results. Ideally, such studies should include the evaluation of both symptomatic and asymptomatic subjects as well as additional biomarker and/or next generation U-LAM detection tests to further enhance the sensitivity of such a combination method to over 95%.
